# High methane ebullition throughout one year in a regulated central European stream

**DOI:** 10.1038/s41598-024-54760-z

**Published:** 2024-03-04

**Authors:** Tamara Michaelis, Felicitas Kaplar, Thomas Baumann, Anja Wunderlich, Florian Einsiedl

**Affiliations:** TUM School of Engineering and Design, Chair of Hydrogeology, Technical University of Munich, Munich, Germany

**Keywords:** Biogeochemistry, Climate sciences, Environmental sciences, Limnology, Biogeochemistry, Climate sciences, Environmental sciences, Limnology

## Abstract

Ebullition transports large amounts of the potent greenhouse gas methane (CH$_4$) from aquatic sediments to the atmosphere. River beds are a main source of biogenic CH$_4$, but emission estimates and the relative contribution of ebullition as a transport pathway are poorly constrained. This study meets a need for more direct measurements with a whole-year data set on CH$_4$ ebullition from a small stream in southern Germany. Four gas traps were installed in a cross section in a river bend, representing different bed substrates between undercut and slip-off slope. For a comparison, diffusive fluxes were estimated from concentration gradients in the sediment and from measurements of dissolved CH$_4$ in the surface water. The data revealed highest activity with gas fluxes above 1000 ml m$^{-2}$ d$^{-1}$ in the center of the stream, sustained ebullition during winter, and a larger contribution of ebullitive compared to diffusive CH$_4$ fluxes. Increased gas fluxes from the center of the river may be connected to greater exchange with the surface water, thus increased carbon and nutrient supply, and a higher sediment permeability for gas bubbles. By using stable isotope fractionation, we estimated that 12-44% of the CH$_4$ transported diffusively was oxidized. Predictors like temperature, air pressure drop, discharge, or precipitation could not or only poorly explain temporal variations of ebullitive CH$_4$ fluxes.

## Introduction

Climate change is no longer a mere scientific phenomenon, but has impacted landscapes, ecosystems and societies around the globe^1^, forcing political action worldwide. To design effective mitigation strategies and adaptation measures, sound carbon budgets and feedback models are essential. To date, greenhouse gas (GHG) emissions from natural aquatic environments are poorly constrained and potential feedback to a warming climate is discussed controversially^2^.

Rivers are one of the largest sources of uncertainty in global methane (CH$_4$) budgets^3^ due to high spatiotemporal heterogeneity and a low number of direct measurements^4^. Yet, rivers and streams have repeatedly been shown to contribute large amounts of this potent GHG to the atmosphere^5–7^, and future emissions may rise further due to warming^8,9^, higher fine sediment inputs^10^, or increased eutrophication^11^. A major transport pathway of CH$_4$ from aquatic environments to the atmosphere, accounting for 50% to 90% of CH$_4$ emissions from lakes^3^, is ebullition, the spontaneous release of gas bubbles from anoxic sediments. In addition, Rocher-Ros et al.^12^ recently suggested that about half of the global CH$_4$ emissions from rivers were emitted via ebullition. However, the contribution of ebullition to riverine CH$_4$ emissions is currently still highly uncertain due to a lack of data especially from mid to high latitudes^13^.

In the context of ebullitive CH$_4$ emissions from rivers, recent literature has mainly focused on reservoir impoundments^14–17^, leaving the relevance of free flowing sections in small streams as GHG emitters less explored. However, headwater streams represent the largest part of most river networks^18^, and several studies suggested an overproportional importance of these environments: McGinnis et al.^19^, as one of the first, demonstrated the potential significance of small streams, showing CH$_4$ emission rates comparable to tropical reservoirs; Castro-Morales et al.^20^ measured 2-7 times higher CH$_4$ partial pressure in tributaries compared to main channels; and Zhang et al.^21^ found an exponential decrease of ebullitive CH$_4$ fluxes with Strahler stream order.

Ebullition is a highly dynamic process and episodic variations may be overseen in short-term observations^22^. Nevertheless, sampling campaigns are often conducted within few days or weeks^23^, or are restricted to spring, summer and autumn for their favorable sampling conditions^19,24^, leaving a lack of winter data from study sites in temperate climates. To the best of our knowledge, only few studies have conducted whole-year investigations on ebullitive CH$_4$ emissions from streams^25,26^.

**Figure 1:**
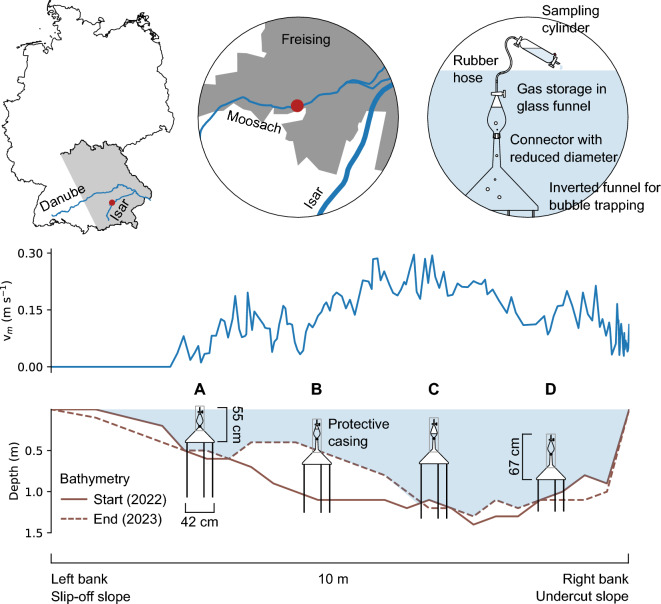
Experimental set-up. A map of the study area is provided in left and center of the top row. The map was compiled with ArcGIS Pro (version 3.0.3). In the middle row, average flow velocity v$_m$ across the stream width is displayed as measured on June 28th, 2022. The cross section with four gas traps is displayed at the bottom. Bathymetry was measured at installation (2022) and removal (2023) of the gas traps. Heavy sedimentation covered parts of sampler B after a period of high flow in spring 2023. A detail in the top right schematically shows the sampling procedure.

This study was designed to collect a comprehensive data set of direct measurements of CH$_4$ ebullition from an anthropogenically impacted small stream and to quantify the GHG emissions throughout the year. Earlier studies have already identified river Moosach in southern Germany as an interesting study site for ebullition due to high CH$_4$ concentrations in the pore-water, a high abundance of methanogens in the hyporheic zone (HZ)^27^, and the detection of gas bubbles in sediment cores^28^. For the present study, bubble traps were installed at four sites in a cross-section located in a river bend, representing different substrates between slip-off slope, central section, and undercut slope (Fig. 1). Measurements of ebullition volumes, CH$_4$ and CO$_2$ concentrations, together with the stable carbon isotopic composition of CH$_4$ ($\delta ^{13}$C–CH$_4$) in the gas bubbles were conducted for 12 months to capture temporal variability of all four seasons. $\delta ^{13}$C-CH$_4$ data are well suited to study conversion processes in the CH$_4$ cycle^27,29,30^. Ebullitive CH$_4$ transport was further compared to estimates of diffusive fluxes across the sediment-water and the water-air interface, calculated from vertical CH$_4$ concentration profiles in the streambed and dissolved CH$_4$ concentrations in the surface water, respectively.

## Materials and methods

### Study site and general setup

The study was conducted at River Moosach, a small stream in the south of Germany which drains a catchment area of 175 km$^2$, including larger peatlands of the Munich Gravel Plain and the Tertiary Hill Country. The river’s course and hydrology have been altered by engineering measures like straightening and the construction of dams and dikes^31^. Nowadays, the river has a very low gradient, in the central section as low as 0.1‰, which leads to an increased accumulation of fines^32^. The organic matter content of the fine fraction was found to be high with an average of 16 $\%$^32^.

To compare ebullitive GHG fluxes from different substrates, a cross section in a river bend was selected. In river turns or meanders, typically a secondary current erodes the outside curve, leading to an undercut slope, and deposits fine bed material at the inside bend, shaping a convex slip-off slope. Incremental differences between the fine deposits of the slip-off slope and the coarser bed substrate at the undercut slope can be studied in these conditions, at equal site characteristics otherwise.

Four bubble traps were permanently installed and sampled over a period of 12 months (Fig. 1). Bubble traps consisted of inverted funnels with an opening diameter of 42 cm, topped with an inverted glass separation funnel with stopcock for gas storage (Fig. S1 in the supplement). Trapping and storage funnels were connected through a rubber stopper with a bore hole reducing the diameter to 14.6 mm. This was meant to hamper convective exchange and thus, reduce transfer of GHGs from the stored gas into the surface water. Bubble traps A (left bank, slip-off slope), B (center left), and D (right bank, undercut slope) were installed on June 8th 2022. Macrophytes grew at sites A and B during July 2022. Therefore, a fourth trap (C, center right) was later installed (July 25th 2022) after discharge and flow velocity measurements to represent the main flow section with highest flow velocities centrally in the river (Fig. 1). All bubble traps were installed approximately 10 to 20 cm above the streambed, such that they were fully submerged during almost all flow conditions. The traps were not designed floating, but provided with legs for fixation in the sediment to avoid tilting with the current, movement in space, or disturbance by animals and people.

### Bathymetry and sediment characterization

The river bathymetry was measured at the days of installation and removal of the gas traps. An echosounder (Deeper, Vilnius, Lithuania) was moved across the river in 0.5 m steps and stream depth was recorded at each interval. Heavy sedimentation occurred at site B after a flooding event in spring 2023 and sampler B was half buried in the deposits after the event (Fig. 1).

For sediment characterization, two sediment cores were taken a little downstream of each bubble trap by manually pushing a liner with 6 cm inner diameter into the sediment. Porosity was measured by weighting sediment cores with a known volume before and after drying at 105 $^\circ$C. Grain size distribution curves were created by sieving with decreasing mesh diameters and sedimentation experiments according to the German norm DIN EN ISO 17892-4. Loss on ignition (LOI) was determined by annealing dried and grinded sediment samples in a furnace at 550 $^\circ$C to constant mass as described in the norm DIN EN 17685-1.

### Flow velocity measurements

Flow velocity measurements were conducted for a relative comparison of the four sites. A velocity distribution over the studied cross section was measured with an Acoustic Doppler Current Profiler (ADCP) (SonTek, Yellow Springs, USA) on June 28$^{th}$, 2022 (Fig. 1). The integrated discharge was with 1.25 ± 0.06 m$^3$ s$^{-1}$ (mean ± standard deviation, n = 5) 34% lower than the average discharge on that day at an official monitoring station 4.5 km downstream (1.68 m$^3$ s$^{-1}$)^33^. Macrophytes covered parts of the cross section during ADCP measurements which may have lead to inaccuracies in the bottom delineation and thus, to an underestimation of the cross sectional area. On the other hand, the official monitoring station was located downstream of the studied cross section behind the confluence with a small diversion creek and thus, there could also be a systematic difference between the two locations.

### Sampling design

Bubble traps were sampled in intervals of 3 to 20 days, depending on expected gas volumes and weather conditions. In total, 158 samples were taken on 66 separate days and analyzed for gas concentrations and carbon stable isotopes of CH$_4$ ($\delta ^{13}$C-CH$_4$). Care was taken not to disturb the sediment during sampling, because extreme ebullition was observed once the sediment was physically stressed. Thus, sampling was carried out from an inflatable dinghy with minimal contact to the bubble traps. For extracting gas from the traps, a sampling cylinder with two glass stopcocks and a septum, pre-filled with de-ionized water, was connected to the inverted glass funnel for gas storage with a water-filled gas-tight rubber-hose (Fig. 1 and S1). By letting water out at the bottom end of the sampling cylinder, gas stored in the bubble trap was sucked into the cylinder. Small amounts of gas up to max. 16 ml (depending on size and filling level of the sampling cylinder) were lost in the rubber hose. Subsequently, all stopcocks were closed and the sampling cylinders were transported to the laboratory for further analysis.

For measurement of dissolved CH$_4$ concentrations in surface water, sampling cylinders were completely filled with stream water. Samples were taken centrally in the cross section approximately 5–10 cm below the water table. In the laboratory, a nitrogen (N$_2$) headspace of 10–20% was created and the sample fixated with NaOH to ensure a pH > 12 and to prohibit microbial turnover.

In addition, geochemical depth-profiles with a 1 cm vertical resolution were measured in the streambed at the beginning of the sampling period at three of the four sites. Three pore-water equilibrium dialysis samplers (peepers) were installed in June 2022, two meter downstream of bubble traps A, B, and D. For a more detailed description of the sampling method see Michaelis et al.^27^. For sampling, peepers were removed from the sediment, cleaned with de-ionized water, and samples withdrawn quickly from the chambers. Samples for CH$_4$ analyses were taken first in 10 ml glass vials previously flushed with synthetic air (80% N$_2$, 20% O$_2$), closed with a rubber-butyl stopper, and prepared with 20 μL 10 M NaOH. A second needle was inserted during sample injection for pressure release while care was taken to reduce turbulence and avoid degassing by slowly filling vials along the walls. For anion- and cation analyses, samples were taken in 1.5 ml glass vials fixated with 10 μL 0.5 M NaOH and 1 M HCl, respectively. All pore-water samples were transported to the laboratory and stored refrigerated until further analysis.

### Laboratory analytics

Gas sampling cylinders for ebullition measurements were first weighted. Gas volumes were determined as weight difference of the completely water-filled gas cylinder and the cylinder with the gas sample. Before further measurements, sampling cylinders were left in the laboratory over night (>12 hrs) for gas equilibration between water phase and headspace at room temperature. Temperature was measured when extracting gas from the cylinder.

CH$_4$ concentrations were measured with the Trace 1300 gas chromatograph with flame-ionization detector (GC-FID) equipped with a TG-5MS column and calibrated with three standards (Rießner Gase, Lichtenfels, Germany). 250 $\mu$L of gas were manually injected three times for each sample. CH$_4$ concentrations in pore-water could be measured directly, but CH$_4$ contents in gas bubbles exceeded the calibrated range of the instrument by far, making a 1:100 dilution necessary. An underestimation of measured concentrations by 4.8$\%$ was quantified with 10 dilutions of a gas concentration standard, each measured in triplicates, and samples were corrected with this factor before further data processing. After dilution and manual injection, concentrations could be obtained with an uncertainty of 5.3$\%$ (relative uncertainty based on 2*SD of all 10 standard dilutions and repeated measurements). All samples were diluted and measured twice to detect and avoid gross errors.

The stable carbon isotope ratio ($\delta ^{13}$C) of CH$_4$ was measured in triplicates with a G2201-i gas analyzer with an analytical uncertainty of < 0.16‰ (Picarro, Santa Clara, USA), calibrated with two standards (Airgas, Plumsteadville, USA). Ebullition samples were further diluted to meet the measurement range specified for the instrument and gas bags with the dilutions were connected to a small sample introduction module (SSIM). For pore-water samples from peeper profiles, vials were directly connected to the SSIM with a needle. Due to the small sample volumes, re-pressurization of the sampling vials with the carrier gas was necessary between repeated measurements.

The high gas volumes and large shares of GHG in ebullition samples allowed analysis of CO$_2$ and N$_2$ contents with a GC Micro Box (SLS Micro Technology, Germany). The instrument was calibrated with two standards (Linde AG, Unterschleißheim, Germany), achieving a relative uncertainty of < 20%.

### Data processing and calculation of ebullitive fluxes

CH$_4$ concentrations in pore-water samples from peepers were determined with the headspace equilibration method^34,35^ adapted for small sample volumes as described previously^27^. Also for ebullition samples it was necessary to determine how the headspace gas composition in the sampling cylinder had changed before the measurements due to partitioning of the gas components between gas and water phases. Initial CH$_4$ and CO$_2$ contents before equiliration were inversely modeled from the measured headspace composition after equilibration using an optimization procedure and the chemical modeling software PHREEQC (version 3.7.3)^36^. The geochemical modeling approach was chosen because it enabled consideration of the lime-carbonic acid balance, which strongly influences CO$_2$ solubility, and because it took into account how the presence of CH$_4$ and CO$_2$ mutually affected the bubble pressure. It was therefore superior to a component-wise calculation with Henry’s law.

Input parameters for each PHREEQC run were gas pressure, temperature in the laboratory during measurements, and a best guess of the gas composition before equilibration. Optimal values for the initial CH$_4$ and CO$_2$ contents were determined by minimizing the sum of squared errors of the measured and modeled final gas composition after equilibration using the python package scipy.optimize (version 1.11.2). Pressure was set to the atmospheric pressure on the sampling day^37^. The calculations were based on the assumption of a full equilibrium between water and gas phases in the sampling cylinder, which can be expected due to a storage time of >  12 hours and shaking. An uncertainty is that changes in pressure due to warming of the sample in the laboratory could not be considered. However, the sensitivity of the inverse modeling method to changes in gas pressure was found to be very low. In 10 random samples, pressure changes by ± 20% only changed final gas contents by < 1%.

Gas content *c* was converted from percent to mol L$^{-1}$ using the molar volume of 22.4 L mol$^{-1}$ and a temperature correction for the surface water temperature on the sampling day T$_{SW}$ in $^\circ$C according to Eq. (1)^34^. Flux F in mol m$^{-2}$ d$^{-1}$ was then calculated by dividing the product of gas content c (mol L$^{-1}$) and gas volume V$_{HS}$ (L) by the area A of the inverted funnel (0.14 m$^2$) and the time difference $\Delta$t (days) since the last sampling (Eq. 2). Volume fluxes were similarly calculated by dividing measured gas volume (V$_{HS}$) by area and time interval.

1 $$\begin{aligned} c (mol/L)= & {} \dfrac{c (\%)}{100} \cdot \dfrac{273}{22.4 (273+T_{SW})} \end{aligned}$$

2 $$\begin{aligned} F= & {} \dfrac{c \cdot V_{HS}}{A \cdot \Delta t} \end{aligned}$$

To address the question of how much CH$_4$ can escape from and how much CO$_2$, N$_2$, and O$_2$ can enter a gas bubble during transport through the water column, we modeled the gas exchange of a pure CH$_4$ bubble with the single bubble dissolution (SiBu) model (version 1.2.6c)^38^. Gas contents in the bubble after passing the water column were estimated for a small (volume V = 0.004 L; diameter d = 2 mm) and large (V = 0.52 L; d = 10 mm) CH$_4$ bubble, a water depth of 1.3 m, and dissolved gas concentrations in the surface water of 0.26 mmol L$^{-1}$ for O$_2$ (measured), 5.2 $\cdot$10$^{-4}$ mmol L$^{-1}$ for CH$_4$ (measured), and 0.15 mmol L$^{-1}$ for CO$_2$ (calculated based on carbonate concentration and a measured pH of 7.9). In addition, we tested how large the gas exchange would be if a medium sized pure CH$_4$ bubble with a volume of 0.02 L (d = 3.4 mm) reached an equilibrium with river Moosach’s surface water (1.3 mmol L$^{-1}$ Na$^+$, 2.5 mmol L$^{-1}$ Ca$^{2+}$, 0.9 mmol L$^{-1}$ Mg$^{2+}$, 1.5 mmol L$^{-1}$ Cl$^-$, 0.3 mmol L$^{-1}$ NO$_3^-$, 0.3 mmol L$^{-1}$ SO$_4^{2-}$, and 11.7 mmol L$^{-1}$ HCO$_3^-$) with PHREEQC^36^.

### Modeling diffusive fluxes across the sediment-water interface

Fluxes across the sediment-water interface were modeled from measured concentration gradients of CH$_4$ in pore-water. A one-dimensional representation of the steady-state diffusion-reaction equation accounting for molecular diffusion, bioturbation, bioirrigation, and a source/sink term (Eq. 3) was solved numerically with the software package PROFILE (version 1.0)^39^.

3 $$\begin{aligned} \dfrac{d}{dz} \left( \phi (D_S + D_B) \dfrac{dC}{dz} \right) + \phi \alpha _i (c_{sw} - c(z)) + R = 0 \end{aligned}$$

where *c*(*z*) is pore-water CH$_4$ concentration as a function of sediment depth z, c$_{sw}$ surface water CH$_4$ concentration, $\phi$ total porosity, D$_S$ molecular diffusivity of CH$_4$ in the sediment, D$_B$ biodiffusivity, $\alpha _i$ the irrigation coefficient, and R the rate of net production or consumption. c$_{sw}$ and $\phi$ were taken from measurements, D$_B$ was assumed to be zero, and R estimated section-wise as model fit parameter with a statistical optimization procedure^39^. D$_S$ was calculated as a function of the molecular diffusivity in water D$_0$ and porosity $\phi$^40^. D$_0$ for a water temperature of 17.0 $^{\circ }$C, measured on the sampling day of the geochemical profiles, was calculated to be 1.49$\cdot$10$^{-5}$ cm$^2$ s$^{-1}$ based on Boudreau^41^. Boundary conditions were a fixed concentration at the top (c$_{sw}$) and bottom of the profile.

Equation (3) does not incorporate an advective flow component and thus cannot take hyporheic exchange into account. The bioirrigation term was used to consider different hyporheic exchange rates between sites. Bioirrigation coefficient $\alpha _i$ was calculated as a function of depth z as suggested by Martin and Sayles^42^ (Eq. 4).

4 $$\begin{aligned} \alpha _i (z) = \alpha _{i,0} \cdot exp(-\alpha _{i,1} x) \end{aligned}$$

Values for parameters $\alpha _{i,0}$ and $\alpha _{i,1}$ were chosen in ranges reported previously^42,43^ such that they represent largest exchange with the surface water in the central river section (site B, $\alpha _{i,0}$ = 170 yr$^{-1}$ and $\alpha _{i,1}$ = 0.4 cm$^{-1}$), medium exchange at the slip-off slope (site A, $\alpha _{i,0}$ = 12 yr$^{-1}$ and $\alpha _{i,1}$ = 0.2 cm$^{-1}$) and very low exchange at the consolidated undercut slope (site D, $\alpha _{i,0}$ = 7 yr$^{-1}$ and $\alpha _{i,1}$ = 1 cm$^{-1}$). Below 10 cm at the center and slip-off slope, or below 5 cm at the undercut slope, $\alpha _i$ was set to zero.

It is worth to be mentioned that the model assumes steady-state conditions. Therefore, short-term dynamics cannot be represented. Since pore-water concentrations measured with dialysis average over a measurement period of several weeks, short-term temporal variations are neither reflected in the data, which makes a quasi steady-state assumption applicable for a first approximation. Nevertheless, interpretation of the modeling results must take these limitations into account.

### Estimation of diffusive fluxes across the water-air interface

The magnitude of diffusive CH$_4$ fluxes across the water-air interface was estimated based on dissolved CH$_4$ concentrations in surface water c$_{sw}$ using Eq. (5)^44,45^.

5 $$\begin{aligned} F_{CH_4} = k \cdot (c_{sw}-c_{air}) \end{aligned}$$

where c$_{sw}$ is the average measured surface water CH$_4$ concentration, c$_{air}$ the background CH$_4$ concentration in the atmosphere, and k the gas transfer velocity in m d$^{-1}$. Values for c$_{air}$ were obtained from the NOAA Global Monitoring Laboratory^46^. An atmospheric CH$_4$ concentration of 1997 ppbv, average of April to June 2021 at the Hohenpeißenberg monitoring station^46^, was the most recent estimate available to represent the sampling season and study area. k was calculated according to Eq. (6)^45,47^.

6 $$\begin{aligned} k = \left( \dfrac{SC_{CH_4}}{600} \right) ^{1/2} / k_{600} \end{aligned}$$

where SC$_{CH_4}$ is the Schmidt number of CH$_4$, and k$_{600}$ the gas transfer rate standardized for a Schmidt number of 600. Following Raymond et al.^45^, we used Eq. (7) for calculating the Schmidt number of CH$_4$ as a function of surface water temperature T$_{sw}$ ($^\circ$C), and Eq. (8) for the estimation of k$_{600}$.

7 $$\begin{aligned} SC_{CH_4} = 1824 - 98.12 \cdot T_{sw} + 2.413 \cdot T_{sw}^2 - 0.0241 \cdot T_{sw}^3 \end{aligned}$$

8 $$\begin{aligned} k_{600} = (v_m \cdot S)^{0.89} \cdot D^{0.54} \cdot 5037 \end{aligned}$$

where v$_m$ is the average stream velocity in m s$^{-1}$ calculated as v$_m$=Q/A with a cross-sectional area A of approximately 10 m$^2$, S the channel slope of 0.15‰^32^, and D the water depth of the stream of 1.3 m at the studied cross section. Daily averaged data for discharge Q (m$^3$ s$^{-1}$) was available from the Bavarian State Office of the Environment^33^. Due to an increase in background CH$_4$ concentrations since 2021 and lower actual discharge than recorded at the official monitoring site (see Sect. "Flow velocity measurements") the estimates for diffusive fluxes across the water-air interface have a tendency to be over- rather than underestimated.

### Estimation of methane oxidation based on stable isotope ratios

CH$_4$ that is transported diffusively through the HZ can be oxidized microbially to CO$_2$ before reaching the water column. Oxidation of CH$_4$ under aerobic and anaerobic conditions leads to an isotopic enrichment in $\delta ^{13}$C-CH$_4$ due to a preferential consumption of lighter isotopes^48^. We used differences in isotopic composition between CH$_4$ in gas bubbles and dissolved CH$_4$ in surface water to estimate the fraction of CH$_4$ oxidized, assuming that gas bubbles represent the source CH$_4$ before oxidation^49^. The fraction oxidized was calculated for an open system at steady state according to Eq. (9)^49,50^.

9 $$\begin{aligned} f_{ox} = \dfrac{\delta _{sw}-\delta _{b}}{(\alpha - 1) \cdot 1000} \end{aligned}$$

where f$_{ox}$ is the fraction of CH$_4$ oxidized, $\delta _{sw}$ and $\delta _{b}$ are the average $\delta ^{13}$C-CH$_4$ values in surface water and gas bubbles, respectively, and $\alpha$ the stable isotope fractionation factor. Isotopic carbon fractionation of CH$_4$ is influenced by transport, here diffusion, and CH$_4$ oxidation ($\alpha = \alpha _{ox}-\alpha _{diff}$)^51^. A wide range of carbon isotope fractionation factors for CH$_4$ oxidation ($\alpha _{ox}$) between 1.003 and 1.039 has been reported, introducing considerable uncertainty into the quantification of CH$_4$ oxidation^50,52^. Stable isotope fractionation factors are not related to the methanotrophic strain or enzymatic pathway^53^, but are strongly temperature dependent^54^. As we have not determined a site specific stable isotope fractionation factor for CH$_4$ oxidation, values for $\alpha _{ox}$ were taken from an environment similar to our study site and corrected by 0.00039 $^\circ$C$^{-1}$^55^ for a median temperature of 12 $^\circ$C (median of measured values between 8 and 16 $^\circ$C). Preuss et al.^51^ reported $\alpha _{ox}$ = 1.031 for saturated soils at 4 $^\circ$C and $\alpha _{diff}$ = 1.001, giving a final $\alpha$ of 1.027 after temperature correction. This value is similar to what has been reported for marine sediments (1.026 to 1.027 at 15 $^\circ$C)^56^. To account for parameter uncertainty we also tested values in the range between 1.017 and 1.037.

### Regression analysis

CH$_4$ fluxes were plotted against several environmental parameters which were named in the literature as potentially affecting CH$_4$ ebullition from streams. Temperature was measured at a monitoring station approximately 630 m upstream of the study site^28^. Daily averaged values for discharge and precipitation were retrieved from the Bavarian State Office of the Environment^33^, air pressure drop was calculated from data made available by the German Weather Service DWD^37^. For each independent variable except for air pressure, an average over the sampling period was calculated and plotted against ebullitive CH$_4$ flux. To correlate the largest pressure drop with CH$_4$ flux, the minimum difference between consecutive air pressure values during a sampling interval was extracted from daily air pressure data. For discharge, precipitation, and air pressure drop, Pearson correlation coefficients were calculated to detect linear correlations. The Pearson correlation coefficient r lies between $-1$ and 1, with values close to zero indicating no correlation, and values close to $-1$1 or 1 a strong negative or positive correlation, respectively. In addition, linear mixed-effects modeling (LMM) was performed with the python package statsmodels (version 0.14.1). For surface water and sediment temperature in 20 cm depth, the modified Arrhenius model (Eq. 10^57^) was fitted as prior employed by Aben et al.^58^ in the context of temperature dependence of CH$_4$ ebullition.

10 $$\begin{aligned} E_T = E_{20} \cdot \theta _s^{(T-20)} \end{aligned}$$

where E$_T$ is the ebullition rate in mmol m$^{-2}$ d$^{-1}$ at temperature T ($^\circ$C), E$_{20}$ the ebullition rate at 20 $^\circ$C, and $\theta _s$ a dimensionless system temperature coefficient. The best fit for E$_{20}$ and $\theta _s$ was determined with the scipy.optimize package (version 1.11.2) in python (version 3.11.4).

## Results

### Sediment characteristics

**Figure 2:**
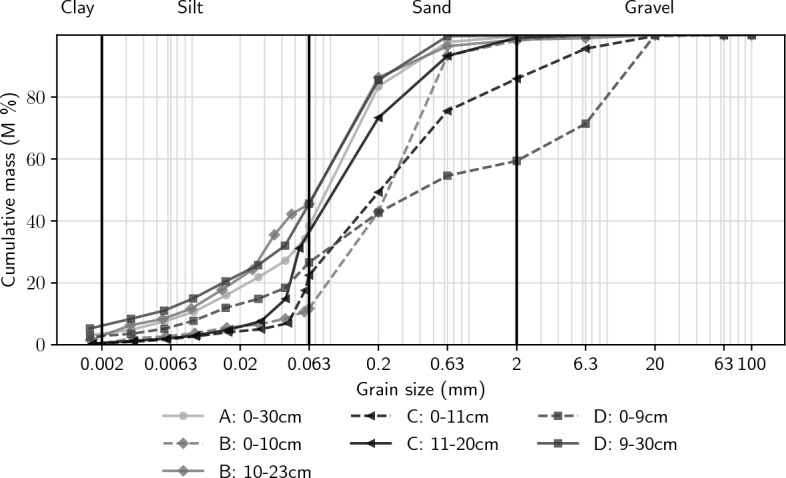
Grain size distribution at the four sampling sites. Sediment cores were taken downstream of each gas trap. Site A represents the slip-off slope, sites B and C the center of the stream, and site D the undercut slope.

Sediment characteristics between the undercut and the slip-off slope were found to differ in the top layer, but were very similar below a depth of approximately 10 cm (Fig. 2). The slip-off slope (site A) was found to have homogeneous fine-grained bed substrate down to at least 30 cm depth, mainly consisting of fine sands and coarse to middle grained silt and an organic carbon content of 14.6% (Tab. S1 in the supplement). Similar material was found throughout the cross section below the top layer. Moving towards the undercut slope, the top layer was inceasingly coarse grained with 87% sand at the center left (site B), 14% gravel, 63% sand at the center right (site C), and 41% gravel, 33% sand at the right bank (undercut slope, site D). LOI was highest at site C with 26.6%, and lowest at site D with 5.0% (above 9 cm depth) to 9.9% (below 9 cm). Porosity was substantially lower at site D (59.9%) compared to the rest of the cross-section (76.5% to 81.0%).

### Vertical pore-water gradients

**Figure 3:**
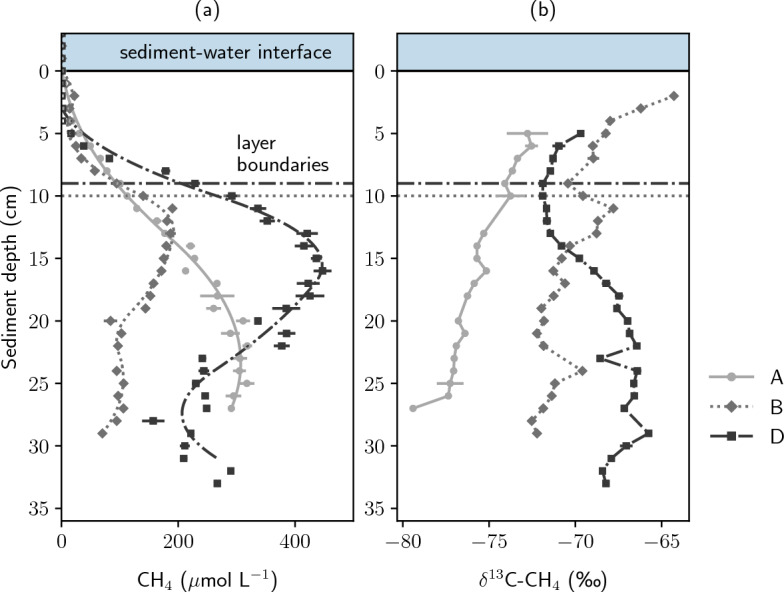
Sediment CH$_4$ concentrations and stable carbon isotopic composition of CH$_4$ in pore water. Site A represents the slip-off slope, site B the center, and site D the undercut slope. In panel (**a**), markers represent measured data and lines modeled CH$_4$ concentrations. Lines in panel (**b**) connect measured values.

Vertical pore-water gradients revealed highest CH$_4$ concentrations between 241 and 447 $\mu$mol L$^{-1}$ at the undercut slope (site D) between 12 to 23 cm depth (Fig. 3). Centrally in the river (site B), CH$_4$ concentrations were lowest with a maximum of 190 $\mu$mol L$^{-1}$. At sites B and D, CH$_4$ concentrations increased close to the sediment layer boundary. Concentrations in the homogeneous deposits at the slip-off slope (site A) increased almost linearly between 1 and 20 cm depth.

$\delta ^{13}$C-CH$_4$ gradients decreased by 0.2 to 0.3‰ per cm with depth at sites A and B, with values at site B being isotopically enriched by approximately 5‰ compared to site A. From 2 to 4 cm depth, a slope change in $\delta ^{13}$C-CH$_4$ was found at site B, showing a pronounced isotopic enrichment by −1.9‰ cm$^{-1}$ towards the top of the streambed, from -68.0‰ in 4 cm to -64.3‰ in 2 cm depth. The $\delta ^{13}$C-CH$_4$ profile measured at site C differed in shape from the other two observations. $\delta ^{13}$C-CH$_4$ was found to be more negative in the top section with an average of − 71.5‰ between 6 to 14 cm depth compared to the bottom of the profile with an average of -67.2‰ from 19 to 33 cm depth.

More information on the geochemistry of the three sites, in particular dissolved O$_2$, anion, and cation measurements, are presented in Fig. S2 in the supplement.

### Ebullition

**Figure 4:**
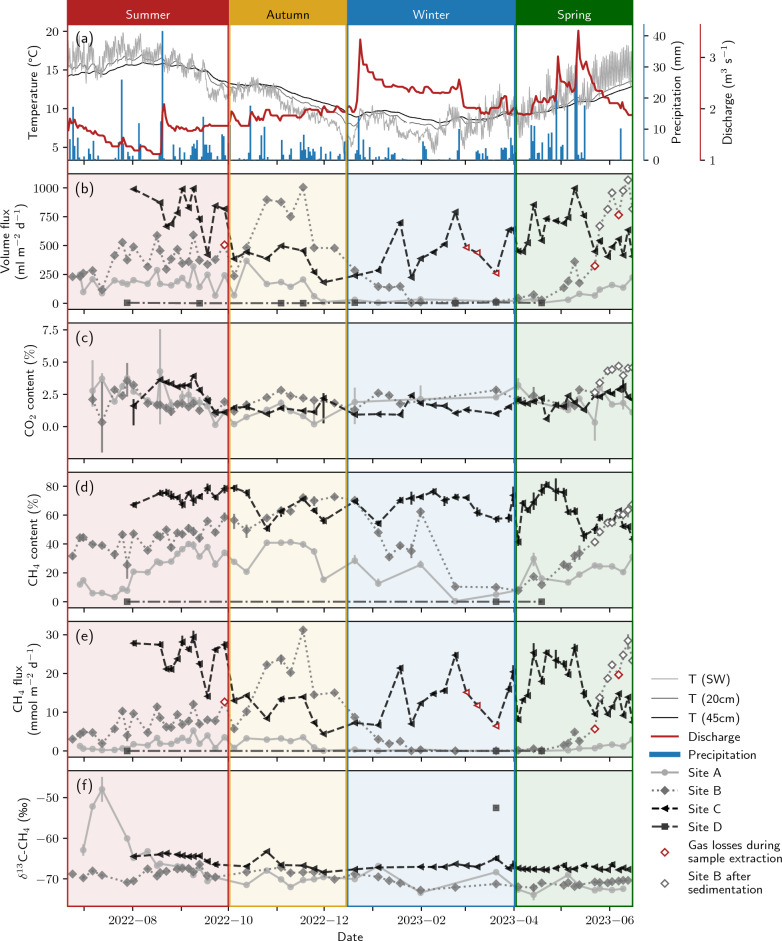
Summary of ebullition measurements. Panel (**a**) shows precipitation, discharge, surface water temperature, and the temperature in 20 cm and 45 cm depth. Gas volume fluxes are displayed in panel (**b**). Panel (**c**) shows CO$_2$ (GC Micro Box) and panel (**d**) CH$_4$ (GC-FID) contents in the gas samples. Two outliers in the CO$_2$ data were removed from the data set of site B (see text). Panel (**e**) depicts CH$_4$ fluxes, and panel (**f**) stable carbon isotopes of CH$_4$. Error bars indicate the range of minimum and maximum measured values for gas contents and the standard deviation of repeated measurements for $\delta ^{13}$C–CH$_4$. For an easier description, the data was grouped into seasons which were chosen based on visual inspection of the data to represent specific seasonal patterns (summer: 15-06-2022 to 01-10-2022, autumn: 01-10-2022 to 15-12-2022, winter: 15-12-2022 to 01-04-2023, spring: 01-04-2023 to 15-06-2023).

Ebullition was observed at all four sites, although only small volume fluxes < 8 mL m$^{-2}$ d$^{-1}$ were detected at site D (Fig. 4). A data summary for the full year is provided in Table 1 and season-specific descriptive statistics are compiled in Tab. S2 in the supplement. During summer, volume fluxes were highest at site C with an average of 802 mL m$^{-2}$ d$^{-1}$, followed by a period in autumn where higher gas volumes were emitted at site B (651 mL m$^{-2}$ d$^{-1}$ at site B compared to 374 mL m$^{-2}$ d$^{-1}$ at site C). Later during winter, volume fluxes at site C increased again to an average of 464 mL m$^{-2}$ d$^{-1}$ while all other sites had low fluxes of less than 35 mL m$^{-2}$ d$^{-1}$ until end of February. Ebullition increased again in May at sites A and B. At site B, volume fluxes went up to a maximum of 1068 mL m$^{-2}$ d$^{-1}$. These spring values at site B were influenced by sedimentation which buried the bottom of the gas trap after a spring flood in mid May (around May 12$^{th}$).

**Table 1:** Data summary. This table shows the main statistical values for the full year period. For ebullition, season-specific data is presented in Tab. S2 in the supplement. For pore-water measurements, one data set represents all values measured in a vertical profile from 1 cm depth onwards.

Data type	Parameter	Site	n	Min	Max	Mean	SD
Ebullition	Volume flux (mL m$^{-2}$ d$^{-1}$)	A	43	3.8	367.4	138.2	91.3
		B	53	2.8	1068.4	409.0	288.4
		C	51	181.4	996.0	581.0	217.6
		D	8	0.0	7.6	2.8	2.7
	CH$_4$ content (%)	A	43	0.4	41.3	23.4	11.8
		B	53	7.7	72.7	44.9	16.4
		C	51	41.4	80.9	66.1	10.1
		D	3	0.01	0.02	0.02	0.01
	CH$_4$ flux (mmol m$^{-2}$ d$^{-1}$)	A	43	0.004	5.3	1.5	1.4
		B	53	0.03	31.2	9.1	8.1
		C	51	4.4	29.4	16.7	7.1
		D	3	1 $\cdot 10^{-5}$	5 $\cdot 10^{-5}$	3 $\cdot 10^{-5}$	2 $\cdot 10^{-5}$
	$\delta ^{13}$C–CH$_4$ (‰)	A	34	− 73.9	− 47.9	− 67.9	5.6
		B	44	− 72.8	− 66.4	− 69.7	1.6
		C	45	− 68.4	− 63.4	− 66.5	1.4
		D	1	−	–	− 52.5	–
Diffusive CH$_4$ flux (mmol m$^{-2}$ d$^{-1}$)	Across air–water interface	–	11	0.21	1.04	0.69	0.24
	Across sediment–water interface	–	3	0.03	0.11	0.06	0.04
Pore–water profiles	CH$_4$ conc. ($\mu$mol L$^{-1}$)	A	27	1.1	318.2	178.6	115.5
		B	28	8.5	190.0	100.6	60.2
		D	33	0.8	447.2	246.9	144.2
	$\delta ^{13}$C–CH$_4$ (‰)	A	20	− 79.4	− 72.5	− 75.6	1.8
		B	26	− 72.5	− 64.3	− 70.0	2.0
		D	28	− 71.9	− 65.8	− 68.8	2.1

CH$_4$ contents of up to 81% were found in the gas bubbles, while CO$_2$ contents remained below 5% with the exception of two outlier values at site B that were removed from Fig. 4. Outliers occurred on February 22$^{nd}$ and April 18$^{th}$, when modeling of measured contents of <1% resulted in final contents of 8% and 18%, respectively. CH$_4$ contents were positively correlated with volume fluxes and generally higher at sites with higher gas emissions (Fig. S3). From June to September 2022, CH$_4$ contents at site A were on average 23% with volume fluxes between 67 and 319 mL m$^{-2}$ d$^{-1}$, at site B 44% with volume fluxes between 116 and 592 mL m$^{-2}$ d$^{-1}$, and at site C 73% with volume fluxes between 420 and 994 mL m$^{-2}$ d$^{-1}$. At site D, gas volumes were generally too low to obtain reliable gas composition or stable isotope measurements. Further, holding times in the storage funnel had to be long in order to reach a sufficient amount of gas for sampling which decreased credibility of measured gas contents and fluxes at site D.

Calculated GHG fluxes were below 2 mmol m$^{-2}$ d$^{-1}$ for CO$_2$, but reached values of up to 31 mmol m$^{-2}$ d$^{-1}$ for CH$_4$. Considering the full year, CH$_4$ fluxes were highest in the central section of the river with a total of 963 mmol CH$_4$ m$^{-2}$ yr$^{-1}$ at site C and 492 mmol CH$_4$ m$^{-2}$ yr$^{-1}$ at site B, followed by the slip-off slope (67 mmol CH$_4$ m$^{-2}$ yr$^{-1}$ at site A). Values were calculated as sum of all measured fluxes at one site and scaled to represent a sampling period of 365 days.

Stable carbon isotopes of CH$_4$ in gas bubbles stayed most of the year below −64‰ (Fig. 4). An exception was July 2022 when $\delta ^{13}$C–CH$_4$ at site A increased up to −47.9‰. Only one value for $\delta ^{13}$C–CH$_4$ at site D could be obtained which was −52.5‰. $\delta ^{13}$C–CH$_4$ values at site B were on average − 3.5‰ more negative than at site C when comparing values measured on equal sampling days. The average $\delta ^{13}$C–CH$_4$ at site B was − 69.7‰ and at site C − 66.5‰. $\delta ^{13}$C–CH$_4$ at site A was more variable than at sites B and C throughout the year.

### Diffusive fluxes and oxidation

**Figure 5:**
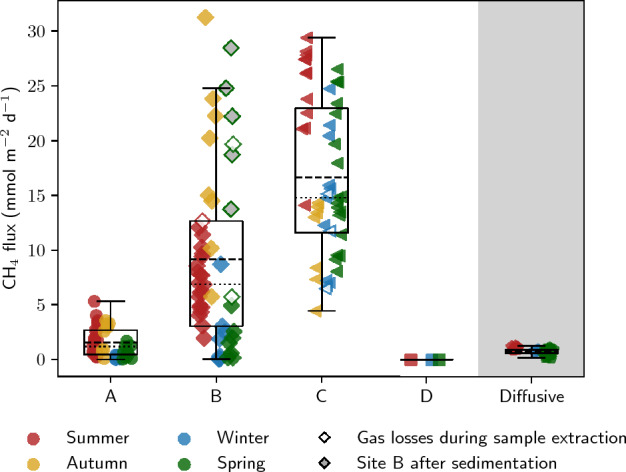
Boxplots showing site-specific differences in CH$_4$ fluxes and a comparison with diffusive fluxes across the water–air interface. Non-filled markers indicate that gas losses occurred during sampling. At site B, gray filling indicates that the gas trap was influenced by sedimentation (spring 2023).

Diffusive CH$_4$ fluxes across the sediment–water interface were modeled to be 0.04 mmol m$^{-2}$ d$^{-1}$ at site A, 0.11 mmol m$^{-2}$ d$^{-1}$ at site B, and 0.03 mmol m$^{-2}$ d$^{-1}$ at site D. Diffusive CH$_4$ fluxes across the water–air interface calculated from dissolved CH$_4$ concentrations in the surface water were higher with an average of 0.69 ± 0.24 mmol m$^{-2}$ d$^{-1}$ and a range between 0.21 and 1.04 mmol m$^{-2}$ d$^{-1}$ (Fig. 5). Based on the assumptions mentioned in Sect. Data processing and calculation of ebullitive fluxes and with a stable isotope fractionation factor of $\alpha$ = 1.027, we calculated that a fraction of 15.9% to 27.8% of this diffusively transported CH$_4$ was oxidized. Lower and higher estimates represent different end member values of $\delta ^{13}$C–CH$_4$ in the gas phase ($\delta _b$ = − 69.7‰, average $\delta ^{13}$C–CH$_4$ at site C, or − 66.5‰ at site B in Eq. (9), respectively). Using the same end members for $\delta ^{13}$C–CH$_4$ and a range of values for $\alpha$ from 1.017 to 1.037 to account for parameter uncertainties, between 44.1% and 11.6% of the CH$_4$ transported diffusively was oxidized.

## Discussion

Gas bubbles were composed of mostly CH$_4$, some CO$_2$, and residual N$_2$. On its way through the water column, a pure CH$_4$ bubble with an initial volume of 0.004–0.52 L would loose 1.7–10.6% CH$_4$ and gain up to 6.9% N$_2$ and up to 3.2% O$_2$ (estimated with the SiBu model^38^). After equilibration with the surface water, a pure CH$_4$ bubble of 0.02 L would reach 7.5% CH$_4$, 20% O$_2$, 72% N$_2$, and 0.1% CO$_2$ (modeled with PHREEQC^36^). These calculations show that exchange processes in the water column cannot explain the final measured gas composition. The measured N$_2$ contents exceeded the values that can occur due to exchange processes during transport and even if an equilibrium were reached, the measured CO$_2$ contents of up to 5% could not be achieved. If the gas exchange with the surface water is limited, the gas composition reflects the conditions at the site of bubble formation^59^. Similar to what has been observed earlier^60^, CH$_4$ contents were positively correlated with volume flux (Fig. S3 in the supplement) and N$_2$ contents followed a reverse pattern. Thus, at higher CH$_4$ production rates, more CH$_4$ and less N$_2$ was found in the gas samples. At very low volume fluxes, the increased surface to volume ratio of the gas sample in the storage funnel and the longer holding times may have further decreased the CH$_4$ content by allowing larger exchange with the surface water before sampling. Interestingly, CH$_4$ and CO$_2$ contents were not correlated (Fig. S3). Since the CO$_2$ contents in the gas samples exceeded the amount that can enter the bubble during exchange with the surface water, CO$_2$ must have been microbially produced in the HZ together with CH4. However, CH$_4$ and CO$_2$ production did not seem to follow the same seasonal fluctuations.

Year-round, CH$_4$ ebullition was highest at the central section of the river bed, represented by sites B and C (Fig. 5). Our initial hypothesis was that highest ebullitive CH$_4$ fluxes were to be expected at the undercut slope (site A), because sediment incubation studies suggested increasing methanogenic potential with decreasing grain size^61,62^, and because higher ebullition rates have been connected to shallow waters^63,64^. However, increased microbial turnover due to higher hyporheic exchange and an increased permeability for gas bubbles in the river’s center may explain the observations. Fischer et al.^65^ measured 2.9 to 5.5 times higher microbial activity in the central river section compared to nearshore habitats. Higher flow velocities in the central channel and a lower penetration resistance of the bed substrate foster the exchange between interstitial and surface water and thus allow better supply of carbon and nutrients from the surface water^32^. An increased carbon supply in the center of the river is supported by very high organic carbon contents at site C (26.6%). In addition, leaf litter and wood were detected in sediment cores from the central streambed.

The interpretation that higher permeability fosters methanogenesis is only valid as long as anoxic conditions prevail, which is unlikely in gravelly river beds where dissolved O$_2$ and other electron acceptors can travel deep into the HZ and prohibit the production of CH$_4$. But once the fine fraction is high enough to ensure anoxic conditions, additional fine material seems to decelerate carbon supply by reducing hyporheic exchange. Flow velocities were lower at the slip-off slope compared to the other sites, so that less allochthonous plant material was transported there, resulting in slower burial of POC with settling sediments. This is in line with intermediate LOI values at site A (14.6%). At the coarse-grained but consolidated undercut slope, plant material could not enter the streambed due to the hard crust atop, as shown by low LOI values (5% and 9%). Surprising in this context was only the low LOI in the top layer of site B (center left) with only 6%. Yet, vertical concentration profiles suggest that the methanogenic zone was located below 10 cm depth, where carbon contents were again much higher (13.3%). The accordance of high sediment organic matter content and high ebullitive CH$_4$ fluxes is in line with results from incubation studies^66,67^.

Higher permeability not only increases carbon and nutrient supply, but also creates pathways for transport of gases to the top of the sediment. We measured highest sediment porosity at the most productive site. But not only the total pore space, but also the size distribution of the pores matters for ebullitive CH$_4$ emissions^68^. In contrast to a presumably small average pore diameter at the homogeneous, fine-grained slip-off slope (site A), leaf litter and wood potentially formed preferential paths for the escape of gas bubbles. Lower pore-water CH$_4$ concentrations centrally in the river bed support the hypothesis that CH$_4$ produced in this area was quickly transported out of the system, removing CH$_4$ from the pore-water. Where the top layer was consolidated (site D), gas could not escape and the pore-water was more enriched in CH$_4$. Dissolved CH$_4$ concentrations in the HZ were below the theoretical threshold for bubble formation of 1.9 mmol L$^{-1}$, which was estimated for in situ conditions with PHREEQC. However, nucleation can occur earlier than predicted by theory due to the catalytic effect of surfaces^69^. In sediments, CH$_4$ bubbles form where the local production rate exceeds the diffusive transport away from the source^68^. We assume that the hetero surface effect and highly localized CH$_4$ production can explain the formation of bubbles in the HZ of river Moosach and that at higher production rates bubbles emerge faster and less CH$_4$ is distributed in the pore space by diffusion.

With regard to the time dimension, ebullition was observed to vary seasonally rather than short-term or randomly. During summer, autumn, and winter periods each sampler showed relatively stable ebullition rates with similar CH$_4$ contents. Seasonal changes were most pronounced at site B. After intermediate fluxes during summer, ebullition increased drastically during autumn, and then declined during winter. The autumn increase could be connected to the end of the vegetation period. Macrophyte roots may have left paths for gas bubbles when dead plants were transported away by the current. After stored gas was released during that period, almost no GHGs escaped until mid-May, when a temperature increase re-activated microbial activity to produce biogenic CH$_4$. Extreme ebullition at site B during spring 2023 may not be comparable with earlier values, because parts of the gas trap were covered in sediment after a discharge peak earlier in the year (see Fig. 1). Fluxes reported after this event need to be interpreted cautiously and we have therefore shown them with white markers in Fig. 4 and grey-filled markers in Fig. 5.

The sustained high CH$_4$ ebullition during winter at site C is especially interesting in comparison with this seasonal behavior of site B prior to being covered. While ebullitive fluxes at B stagnated at very low rates during winter, they even increased at C despite the cold water temperatures. A sudden release of gas produced during summer seems unlikely here, because fluxes would be expected to drop after this event if no new CH$_4$ is produced in the methanogenic zone. We speculate that only active methanogenesis during the winter period may explain the high gas volumes and bubble CH$_4$ contents during winter and early spring seasons at site C. This is surprising since CH$_4$ ebullition has been shown to be a highly temperature-dependent process^58^. However, some strains of methanogens are known to inhabit cold environments in high altitudes and latitudes^70,71^. The consistent difference in $\delta ^{13}$C–CH$_4$ of 3.5‰ between sites B and C indicates that the proportions of methane-forming pathways are different in the two stream sections, possibly due to the availability of different substrates. Methanol-derived methanogenesis, a cold-adapted production pathway used by psychrophilic methanogens^70,72,73^, could have played a larger role at site C than in the rest of the river bed. The methylotrophic methanogens *Candidatus* “Methanomethyliales” (phylum *Candidatus* “Verstraeteaechaeota”) and Methanomassiliicoccales, the latter associated with CH$_4$ production from methanol in freshwater wetlands^74^, have been detected in the bed of river Moosach^27^ which may support this hypothesis.

Values of $\delta ^{13}$C–CH$_4$ not only showed differences between the sites but also changes over time. At site A, an isotopic shift towards heavier isotopes occurred concurrently with the main plant growth period in July. Two mechanisms could have caused this effect. Microbial oxidation can increase the content of $^{13}$C in the remaining CH$_4$ due to preferential consumption of the lighter isotope $^{12}$C, but CH$_4$ transported as gas bubbles is assumed to escape oxidation due to the high transport velocity. More likely, the shift in CH$_4$ isotopic composition reflects a change in the main methanogenic pathway, for example a higher contribution of acetoclastic methanogenesis during this period. Acetoclastic methanogenesis is associated with less negative $\delta ^{13}$C–CH$_4$ (− 50‰ to − 60‰) than hydrogenotrophic methanogenesis (− 60‰ to − 110‰) or methane production from methanol^75^. Plant growth during that time of the year could have influenced the availability of substrates or the isotopic composition of the substrates.

**Figure 6:**
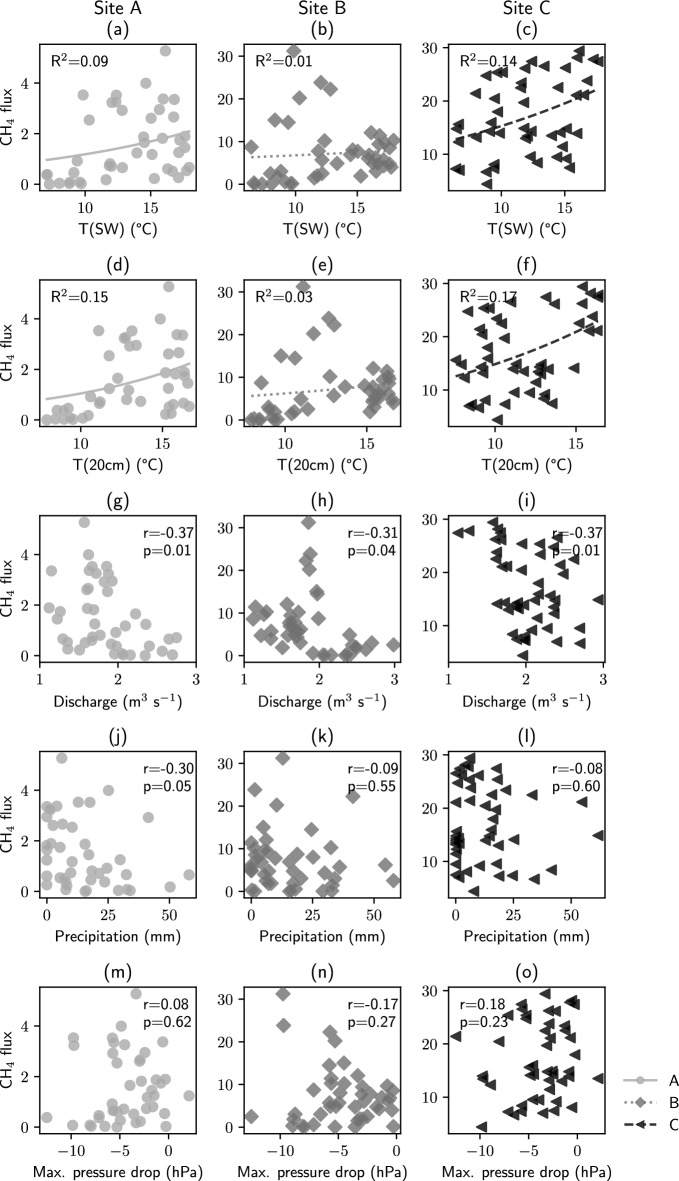
Regression analysis of methane fluxes and relevant parameters. Precipitation represents the sum, all other parameters the average during the gas collection time. For surface water and sediment temperature, the modified Arrhenius model was fitted. For discharge, precipitation, and air pressure drop, Pearson correlation coefficients were calculated to test for linear regression. Correlations were statistically significant ($p<$ 0.05) only for discharge. There was also a statistically significant correlation between temperature (both T$_{SW}$ and T$_{20cm}$) and CH$_4$ flux at sites A and C (Pearson correlation). At site B, high spring fluxes were not considered due to the reduced comparability after a sedimentation event, which partly buried the gas trap.

To explain the observed seasonal dynamics, we have tested how well the modified Arrhenius model can describe the relation of ebullition and temperature, and if a linear correlation between CH$_4$ flux and discharge, precipitation, or air pressure drop can be found with Pearson correlation coefficients (Fig. 6). A strong non-linear temperature-dependence of ebullitive CH$_4$ fluxes from aquatic ecosystems to the atmosphere has been observed earlier^58^. Other studies suggested that CH$_4$ ebullition from streams can be flow-dependent^19^ and that air-pressure drops, for example during storms, can induce ebullition, while high pressure inhibits ebullition^64,76^.

For our data, no or only low correlation was found for air pressure drop and precipitation (|r| $\le$ 0.30). Discharge showed a statistically significant moderate negative correlation (r between − 0.31 and − 0.37). Similarly, LMM of the full data set with site as a random factor was statistically significant for temperature (p$_{T(SW)}$ = 0.013; p$_{T(20cm)}$ = 0.001) and discharge (p $< 0.001$) but not for precipitation (p = 0.285) or pressure drop (p = 0.808). There was a linear negative correlation between temperature and discharge because discharge during the winter is generally higher at river Moosach than during summer. The increased CH$_4$ fluxes at lower discharge may therefore be either season or flow dependent. To summarize, precipitation and air pressure drop do not seem to be good predictors for CH$_4$ emissions while temperature and discharge appear to have a potentially interrelated influence on CH$_4$ ebullition.

The modified Arrhenius model only yielded a very poor fit with generally low R$^2$ values and could not well describe the relation between CH$_4$ ebullition and temperature. The poor quality of fit indicates that the model is not well suited to describe the temperature-depenence of CH$_4$ ebullition and that temperature alone is insufficient to predict the amount of CH$_4$ released to the atmosphere. Other factors, especially site characteristics and sediment properties, must be considered when extrapolating from point measurements. Pronounced differences in volume fluxes and CH$_4$ contents were found between the sites, and four sites were insufficient for a sound statistical evaluation or extrapolation. For example, fluxes at site D were year-round way below all other sites, and winter fluxes at site C were on average 7 times larger than summer fluxes at site A. Overall, spatial heterogeneity in CH$_4$ ebullition was considerably larger than temporal variability which is in line with some other studies^24,77^. For future sampling campaigns, we would, therefore, recommend to focus on the representation of as many sampling sites as possible, as opposed to monitoring one site over a longer time. A large number of field sites, where both diffusive gase transport and ebullition effects are monitored, and a solid bed substrate characterization are necessary for reliable budgeting.

Estimated diffusive fluxes across the sediment-water interface between 0.03 and 0.11 mmol m$^{-2}$ d$^{-1}$ were lower than the average 0.69 mmol m$^{-2}$ d$^{-1}$ calculated for fluxes across the water–air interface. The first could be underestimated, because a stronger exchange with surface water may exist than the model could represent without an advective flow component. This advective flow would dilute CH$_4$ concentrations at the top of the streambed and transport this CH$_4$ into the surface water. Also the assumption of steady state is a drawback of the applied model and could lead to an underestimation of the actual diffusive fluxes across the sediment-water interface. Another reason for the lower fluxes across the sediment-water interface compared to the water− air interface could be the supply of CH$_4$ to the surface water from rising gas bubbles. Overall, we consider CH$_4$ flux estimates across the water− air interface as more reliable for total CH$_4$ emissions than flux estimates across the sediment− water interface and therefore, only consider the first in the following comparison.

Ebullitive CH$_4$ fluxes were up to 30 times larger than diffusive fluxes. This is in line with global estimations for lakes^3^, but contradicts what has been described for rivers recently^47^. In fact, ebullitive CH$_4$ fluxes measured in this study were higher than in many other stream systems. Stanley et al.^4^ reported average CH$_4$ ebullition fluxes of 1.96 mmol m$^{-2}$ d$^{-1}$ (ranging from 0.0 to 9.4 mmol m$^{-2}$ d$^{-1}$), and Robison et al.^24^ 1.00 ± 0.23 mmol m$^{-2}$ d$^{-1}$ (ranging from 0.01 to 1.79 mmol m$^{-2}$ d$^{-1}$). Ebullition in river Moosach reached rates typically found in tropical reservoirs, similar to what was measured by McGinnis et al.^19^ (21.3 mmol m$^{-2}$ d$^{-1}$). On a global scale, a linear relation between diffusive and ebullitive CH$_4$ fluxes has recently been used for estimating total riverine CH$_4$ emissions, leading to roughly equal contributions of both processes^12^. Our data challenges the assumption that it is possible to infer ebullition rates from diffusive flux estimates. It needs to be said that Rocher-Ros et al.^12^ had very robust data on diffusive CH$_4$ fluxes but had to rely on very scarce data on CH$_4$ ebullition for this global estimate (>24000 measurements of dissolved CH$_4$ concentrations plus >8000 direct measurements of diffusive CH$_4$ fluxes versus only 630 observations of ebullition). Obviously, the data available on CH$_4$ ebullition is insufficient, especially considering the high spatiotemporal variability of this process, as also documented in the present manuscript.

Reasons for the untypically high ebullition rates we measured could be the very low gradient and the large amount of fine deposits in the stream. Both are related to anthropogenic alterations like the construction of a series of dams^32^. The controlled discharge conditions prohibit larger disruptions of the streambed and therefore allow stable methanogenic communities to establish. An additional input of carbon and nutrients not only from the nearby peatlands, but also from agricultural fields around the river may further foster turnover rates in the HZ, particularly after heavy precipitation events and floods. Crop lands have been shown to significantly raise erosion rates^78^. One might therefore speculate that human influence could have an enhancing effect on CH$_4$ emissions from small streams. We did not specifically test for anthropogenic effects on CH$_4$ ebullition and their magnitude remains an open question for future research. But if indeed land-use changes and anthropogenic alterations of river Moosach’s course and hydrology have enhanced the CH$_4$ emission potential, renaturation measures at river Moosach could be beneficial from a climatological perspective.

Stream sediments have repeatedly shown a potential for aerobic as well as anerobic oxidation of CH$_4$^61,62,79^. CH$_4$ oxidation would be expected in a narrow zone at the oxic-anoxic interface if CH$_4$ is transported diffusively^80^. However, if CH$_4$ mainly escapes to the atmosphere in the form of gas bubbles, it most likely escapes microbial oxidation. In river Moosach, a potential for CH$_4$ oxidation coupled to O$_2$ reduction and denitrification was found earlier^27^. In this study, we calculated that up to 44% of the CH$_4$ transported diffusively was oxidized, but again it should be mentioned that the diffusive pathway was of minor importance. Of the three geochemical profiles, only site B showed a clear isotopic enrichment in $\delta ^{13}$C− CH$_4$ together with a decline in CH$_4$ concentrations towards the top of the streambed (Fig. 3). At site D, a decrease in dissolved CH$_4$ concentrations combined with an enrichment in $\delta ^{13}$C− CH$_4$ below 15 cm depth could either be caused by CH$_4$ oxidation or mixing processes. As dissolved O$_2$, NO$_3^-$, and SO$_4^{2-}$ were all consumed above 9 cm (Fig. S2), oxidation seems rather unlikely. However, we cannot exclude that trace amounts of dissolved O$_2$ triggered CH$_4$ oxidation. Also iron and manganese remain as potential electron acceptors although their environmental relevance is often limited by their usually low bioavailability^81^. On the other hand, a concurrent decrease in NH$_4^+$ concentrations below 15 cm depth suggests a lower availability of reactive carbon because NH$_4^+$ is released during organic matter decomposition^82^. A combination of lower methanogenesis rates and a shift in the relative contribution of different production pathways, which are linked to different $\delta ^{13}$C− CH$_4$ values, are alternative explanations of the observations. Reduced carbon supply compared to the other sites due to the consolidation of the top layer is a likely reason. In the shallow, well-mixed stream, there is also no significant CH$_4$ oxidation potential in the water column as would be expected in deep lakes^29,30^. For our study site, where fine sediment dominates the river bed, we can therefore conclude that CH$_4$ oxidation is not too relevant as a CH$_4$ sink.

## Conclusions

Ebullition was monitored at four sites in one cross section of a small stream over the course of a full year. Spatial heterogeneity of the HZ was large and CH$_4$ fluxes differed strongly between sites. Ebullitive CH$_4$ fluxes were up to 30 times larger than diffusive CH$_4$ fluxes. Year-round, the central section of the river bed emitted most CH$_4$ as gas bubbles. Reasons are probably a good supply of carbon and nutrients due to a higher potential for exchange with the surface water, and a higher permeability of the sediment for gas bubbles. Gas fluxes varied with the four seasons rather than in short term or random intervals. At one site, a sustained high ebullitive CH$_4$ flux during winter demonstrated CH$_4$ production in the HZ even at cold water temperatures down to < 8 $^\circ$C. In comparison with diffusive fluxes, ebullition transported up to 30 times more CH$_4$ to the atmosphere and just 12% to 44% of the CH$_4$ transported diffusively was oxidized.

### Supplementary Information

Supplementary Information.

## Data Availability

The datasets generated and analysed during the current study have been uploaded to a figshare repository and can be accessed under  10.6084/m9.figshare.24298354.
