# Author Correction: High methane ebullition throughout one year in a regulated central European stream

**DOI:** 10.1038/s41598-024-57907-0

**Published:** 2024-03-27

**Authors:** Tamara Michaelis, Felicitas Kaplar, Thomas Baumann, Anja Wunderlich, Florian Einsiedl

**Affiliations:** TUM School of Engineering and Design, Chair of Hydrogeology, Technical University of Munich, Munich, Germany

Correction to: *Scientific Reports* 10.1038/s41598-024-54760-z, published online 04 March 2024

The original version of this Article contained a repeated error. The reported isotope values in per mill (‰) were incorrectly given as in per cent (%) throughout the article.

The original Article has been corrected.

