# Supplementary material for: High methane ebullition throughout one year in a regulated central European stream
Source: Sci Rep. 2024 Mar 4;14:5359. doi: 10.1038/s41598-024-54760-z (PMC11310342; doi:10.1038/s41598-024-54760-z)
Supplement: Supplementary file 1 — Supplementary Information. [file 41598_2024_54760_MOESM1_ESM.pdf]

Supplement to

High methane ebullition throughout one year in a regulated central European stream

Tamara Michaelis, Felicitas Kaplar, Thomas Baumann, Anja Wunderlich, und Florian Einsiedl*

Technichal University of Munich, TUM School of Engineering and Design, Chair of Hydrogeology, Munich, Germany

*f.einsiedl@tum.de

S1 Photographs of the gas traps and sampling procedure

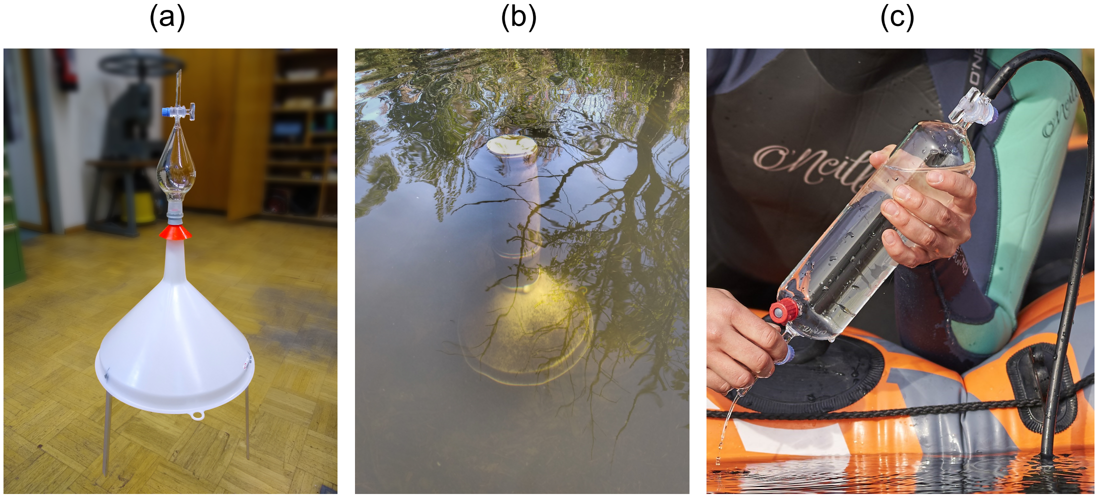

Figure S1. Photographs of the gas traps and sampling procedure. Panel (a) displays a gas trap in the workshop. Legs were later extended to at least 60 cm. Panel (b) shows a sampler after installation in river Moosach. The distance between the bottom of the gas trap and the streambed was at least 10 cm at the day of installation. The sampling procedure is illutrated in panel (c). Pictures were taken by Felicitas Kaplar and Julia Bergmeister.

S2 Additional values and descriptive statistics

Quantitative information on the sediment characteristics is given in Tab. S1. Descriptive statistics of ebullition data for each season are summarized in Tab. S2.

Table S1. Sediment characteristics at the four sampling sites. Grain size distribution is given in percent gravel, sand, and silt/clay fractions. Porosity n and loss on ignition (LOI) are also given in percent.

Site	Depth (cm)	Gravel (%)	Sand (%)	Silt & Clay (%)	n (%)	LOI (%)
A	0-30	0.4	61.3	38.3	78.0	14.6
B	0-10	1.7	86.6	11.7	76.5	6.0
	10-23	1.5	53.1	45.4		13.3
C	0-11	14.1	63.5	22.4	81.0	26.6
	11-20	0.8	65.3	33.9		
D	0-9	40.6	32.8	26.6	59.9	5.0
	9-30	0.2	54.2	45.6		9.9

Table S2. Summary of ebullition data grouped by season.

Parameter	Site	Summer					Autumn					Winter					Spring				
		**n**	**min**	**max**	**mean**	**SD**	**n**	**min**	**max**	**mean**	**SD**	**n**	**min**	**max**	**mean**	**SD**	**n**	**min**	**max**	**mean**	**SD**
Volume flux ($mL\,m^{-2}\,d^{-1}$)	A	20	66.8	319.4	187.1	63.9	8	10.9	367.4	150.9	110.7	5	5.9	34.0	22.8	11.2	9	3.8	158.6	72.6	54.8
	B	22	116.2	591.9	378.4	120.5	8	235.9	1004.7	650.7	269.1	8	2.8	284.6	93.5	101.9	14	28.9	1068.4	470.2	386.1
	C	12	420.3	994.4	801.7	164.5	7	181.4	496.7	374.1	111.7	13	223.7	790.8	464.4	185.9	18	403.8	996.0	608.1	167.1
	D	2	0.4	4.1	2.3	2.7	2	1.0	1.3	1.1	0.2	3	0.0	7.6	4.2	3.9	1	-	-	2.7	-
CH_4 content (%)	A	20	3.1	40.0	23.0	12.4	8	15.3	41.3	32.7	10.2	5	0.4	28.5	14.4	12.4	9	8.0	29.8	20.1	6.8
	B	22	25.4	58.5	43.9	7.8	8	49.5	72.7	63.1	8.2	8	10.0	70.3	38.2	21.8	14	7.7	63.5	38.3	19.2
	C	12	67.1	78.7	73.2	3.7	7	50.6	78.9	65.5	10.3	13	54.1	76.4	67.7	7.3	18	41.4	80.9	61.6	11.4
	D	1	-	-	0.02	-	0	-	-	-	-	1	-	-	0.02	-	1	-	-	0.01	-
CH_4 flux ($mmol\,m^{-2}\,d^{-1}$)	A	20	0.2	5.3	1.9	1.4	8	0.07	3.5	2.2	1.3	5	0.004	0.4	0.2	0.2	9	0.03	1.7	0.7	0.6
	B	22	1.9	12.7	7.1	2.9	8	5.7	31.2	17.9	8.2	8	0.03	8.7	2.1	2.9	14	0.1	28.5	10.4	10.4
	C	12	14.1	29.4	24.6	4.3	7	4.4	14.3	10.7	3.9	13	6.4	27.7	13.8	6.0	18	8.1	26.5	16.3	6.1
	D	1	-	-	$3 \cdot 10^{-5}$	-	0	-	-	-	-	1	-	-	$5 \cdot 10^{-5}$	-	1	-	-	$1 \cdot 10^{-5}$	-
$\delta^{13}C$-CH_4 (‰)	A	15	-70.6	-47.9	-64.1	6.4	7	-72.0	-68.0	-70.2	1.3	4	-73.4	-66.8	-69.7	2.8	8	-73.9	-69.0	-72.2	1.4
	B	17	-70.9	-66.4	-68.6	1.2	6	-70.1	-67.2	-68.5	0.9	7	-72.8	-68.8	-70.9	1.4	14	-72.2	-70.4	-71.1	0.6
	C	10	-66.5	-63.7	-64.6	0.8	6	-68.4	-63.3	-66.6	1.7	11	-67.7	-65.0	-66.8	0.7	18	-67.8	-66.5	-67.4	0.4
	D	0	-	-	-	-	0	-	-	-	-	1	-	-	-52.5	-	0	-	-	-	-

S3 Complete geochemical profiles

In addition to CH_4 concentrations and $\delta^{13}C$-CH_4, dissolved O_2, anion, and cation concentrations were measured in pore-water samples (Fig. S2). Dissolved O_2 concentrations were measured with a Clark-type microsensor (Unisense, Aarhus, Denmark) by piercing the membrane of the peeper chambers immediately upon retrieval from the streambed and cleaning with deionized water. Samples for anion and cation concentration measurements were taken in 1.5 ml glass vials and analyzed with a system of two ICS-1100 ion chromatographs (Thermo Fisher Scientific) with Dionex IonPac^{TM} columns AS9-HC for anions and CS12A for cations.

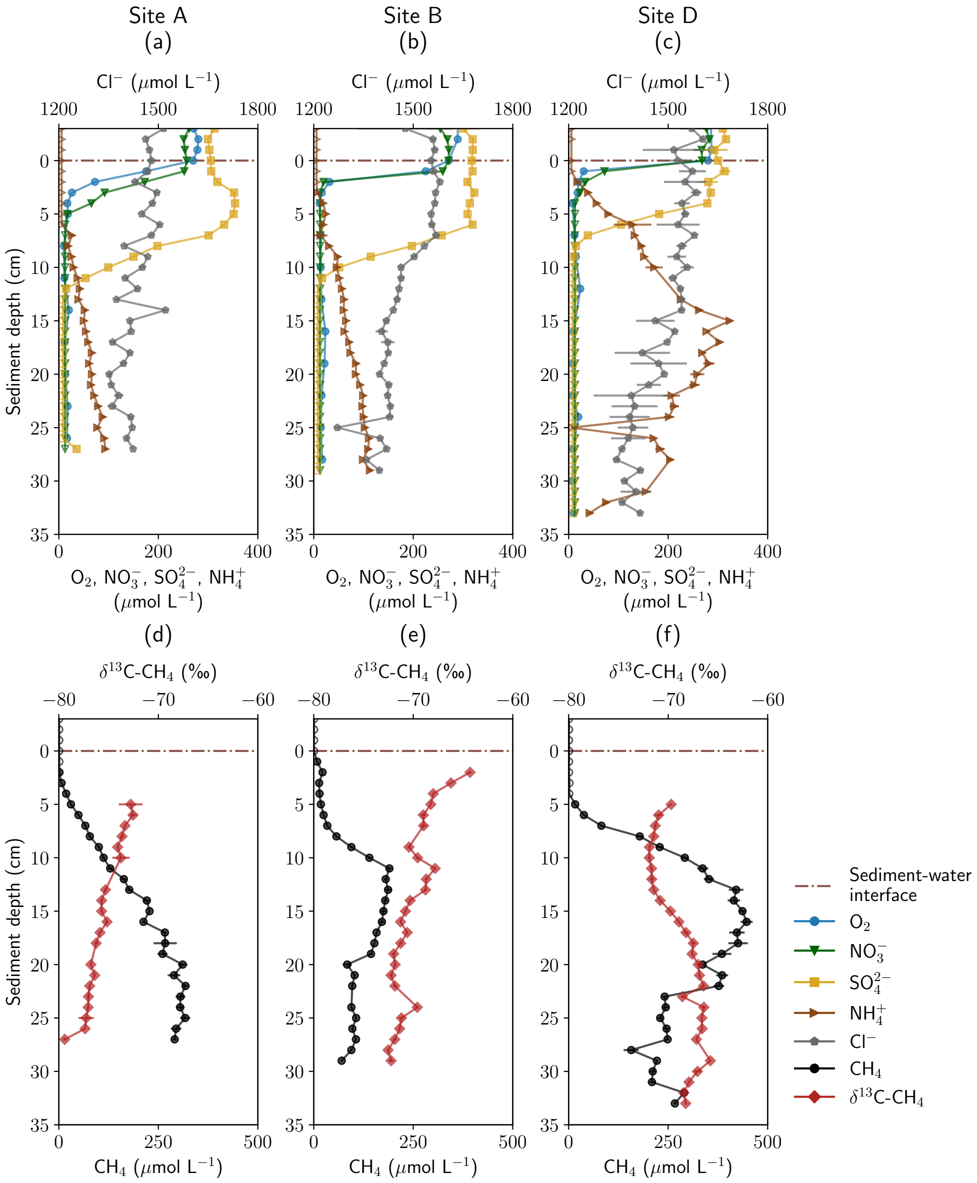

Figure S2. Additional data on geochemical profiles. Error bars indicate standard deviations of triplicate measurements. Markers of measurements below the limit of detection are not filled.

S4 Cross correlations between variables

The relation between CH_4 content and volume flux, CH_4 content and $\delta^{13}C$-CH_4, and CH_4 and CO_2 contents were tested by calculating the Pearson correlation coefficient. A statistically significant correlation was detected between CH_4 content and volume flux, whereas neither CH_4 content and δ^{13}C-CH_4 nor CH_4 and CO_2 contents were linearly correlated. Figure S3 displays both scatter plots twice, once highlighting the site and once the season.

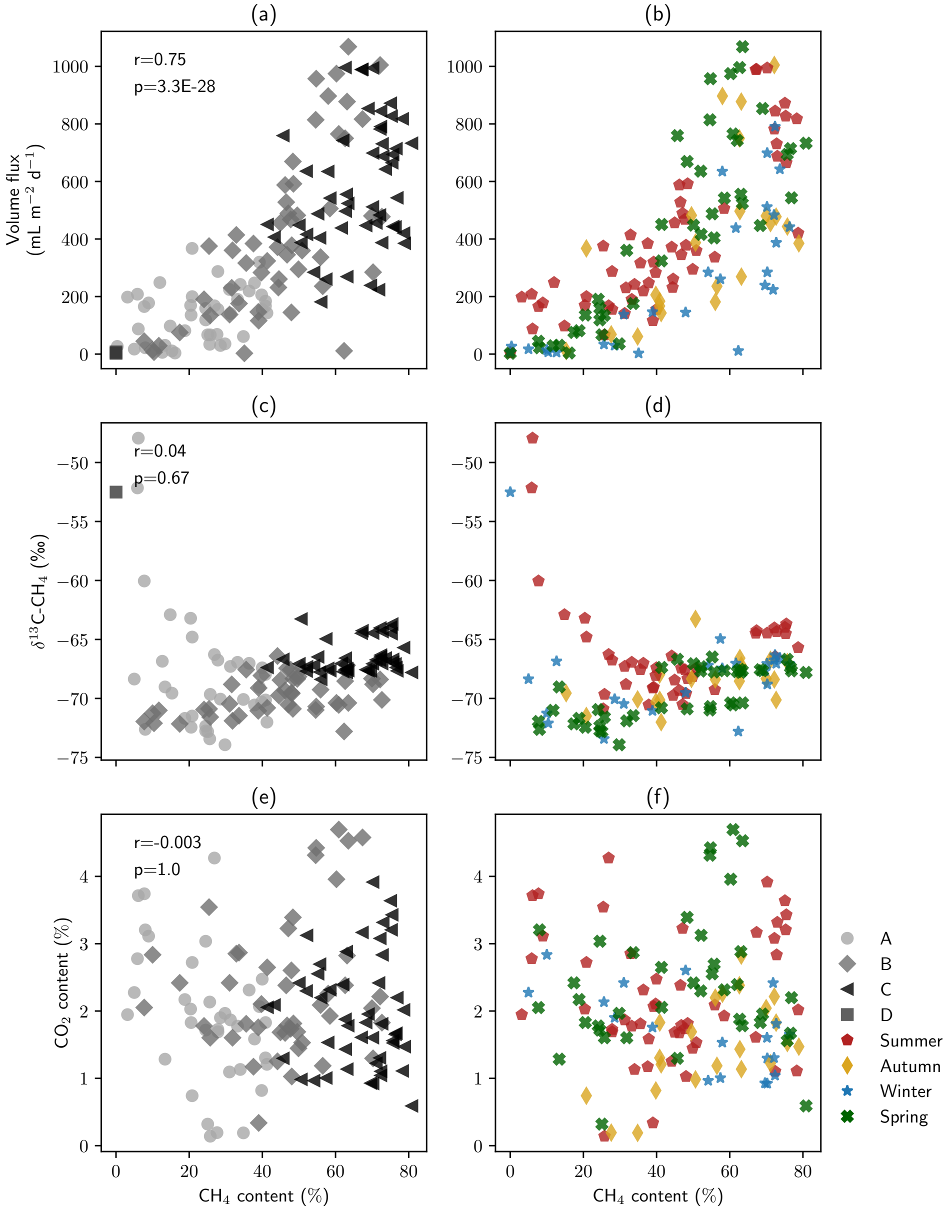

Figure S3. Cross correlations between measurements. Panels (a), (c), and (e) are colored by site, panels (b), (d), and (f) by season. Panels (a) and (b) display CH_4 content and volume flux, panels (c) and (d) CH_4 content and $\delta^{13}C$-CH_4, and panels (e) and (f) CH_4 and CO_2 contents. Peason correlation coefficients and p-values are shown for all data. A statistically significant correlation ($p < 0.05$) was found for CH_4 content and volume flux.
